# Unveiling systemic responses in kidney transplantation: interplay between the allograft transcriptome and serum proteins

**DOI:** 10.3389/fimmu.2024.1398000

**Published:** 2024-07-16

**Authors:** Konrad Buscher, Rebecca Rixen, Paula Schütz, Veerle Van Marck, Barbara Heitplatz, Gert Gabriels, Ulrich Jehn, Daniela Anne Braun, Hermann Pavenstädt, Stefan Reuter

**Affiliations:** ^1^ Division of General Internal Medicine, Nephrology and Rheumatology, Department of Medicine D, University Hospital of Münster, Münster, Germany; ^2^ Institute of Pathology, University Hospital of Münster, Münster, Germany

**Keywords:** kidney, kidney transplantation, systems biology, allograft, transcriptomics

## Abstract

Immunity, as defined by systems biology, encompasses a holistic response throughout the body, characterized by intricate connections with various tissues and compartments. However, this concept has been rarely explored in kidney transplantation. In this proof-of-concept study, we investigated a direct association between the allograft phenotype and serum protein signatures. Time-matched samples of graft biopsies and blood serum were collected in a heterogeneous cohort of kidney-transplanted patients (*n* = 15) for bulk RNA sequencing and proteomics, respectively. RNA transcripts exhibit distinct and reproducible, coregulated gene networks with specific functional profiles. We measured 159 serum proteins and investigated correlations with gene expression networks. Two opposing axes—one related to metabolism and the other to inflammation—were identified. They may represent a biological continuum between the allograft and the serum and correlate with allograft function, but not with interstitial fibrosis or proteinuria. For signature validation, we used two independent proteomic data sets (*n* = 21). Our findings establish a biological link between the allograft transcriptome and the blood serum proteome, highlighting systemic immune effects in kidney transplantation and offering a promising framework for developing allograft-linked biomarkers.

## 1 Introduction

Monitoring and maintaining renal function after renal transplantation (RTx) poses a challenge for physicians. The main reason is that graft damage is often detected late using routinely monitored parameters such as serum creatinine or albuminuria (1, 2). While ultrasound examination is crucial in the clinical workup of graft dysfunction, its sensitivity is low compared to cellular or molecular assessments (3). Donor-specific antibody (DSA) monitoring is expensive, requires several days to obtain results, and does not reliably prove humoral rejection (4, 5). Many promising serum and urine biomarkers, including donor-derived cell-free DNA, have been thoroughly studied, but several drawbacks hampered the clinical implementation as routine parameters (6–10). Consequently, the renal biopsy remains the preferred method to determine the immunological status of the graft (11). However, many centers have abandoned protocol biopsies due to their invasive nature, risk to the graft, low patient comfort, and potential for misinterpretation and sampling error (12). To date, even well-validated scores with functional, histological, and immunological parameters have not yet found widespread clinical application (13). Together, there is an unmet need for new diagnostic ways to assess allograft immunity during RTx.

Systems biology investigates the interconnected nature of biological responses across different tissues and organs (14). It leverages the fact that the immune system is decentralized and relies on various pathways to mount effective responses. Ideally, a systems framework integrates the simultaneous measurement of large data across many tissues and dimensions, such as the epigenome, transcriptome, metabolome, microbiome, T-cell receptor repertoires, and proteome. Supported by successful applications in other medical fields (15, 16), it promises to overcome imperfect gold standards, optimize clinical phenotyping, identify new biomarkers, and eventually improve allograft outcomes (17, 18). Great progress has been made in the Kidney Precision Medicine Project, which targets chronic kidney disease (CKD) and acute kidney injury using multidimensional phenotyping and computational modeling (18, 19). In RTx, many omic technologies have been used to identify new gene signatures or biomarkers (20). A holistic perspective advocated by systems biology is only beginning to be adopted, and large RTx frameworks are missing (20). In this study, we hypothesized that the serum proteome is interconnected with the allograft via systemic immune responses. Therefore, we investigated a biological link between the local allograft gene landscape and peripheral serum protein signatures using a data-driven and unsupervised approach.

## 2 Methods

### 2.1 Patient selection and ethics

Patients with an indication for a graft biopsy were included with the following criteria: acute kidney injury as defined by KDIGO without obvious cause, and/or persistent increase in proteinuria without obvious cause at the discretion of the attending physician. The induction therapy was basiliximab, or antithymocyte globulin. The current immunosuppressive regimen consists of tacrolimus (Prograf), mycophenolate (CellCept/Myfortic), and prednisolone (Solu-Decortin H/Decortin H). Cyclosporine A (Sandimmun) and/or everolimus (Certican) were taken in a few cases. The exclusion criteria were coinfections. The three data sets (training set, validation set 1, and validation set 2) were independently collected and analyzed (details in **Supplementary Table S1**). The training set includes RNA sequencing data matched with serum proteomics, whereas the other two data sets only include serum proteomics. We did not merge these data to avoid batch effects. All patients consented to the study approved by the Ethics Committee (“Ärztekammer Westfalen-Lippe and Universität Münster”; No. 2011-400-f-S and No. 2019-337-f-S). The study was carried out according to the Declaration of Helsinki.

### 2.2 Biopsy scoring and patient data

Biopsies were graded according to the Banff classification (11). No Banff score was available for biopsies with less than seven glomeruli, according to specimen adequacy definitions. Here, fibrosis was measured using a similar classification system and labeled as IFTA grades 0–3. In addition, for each patient, the following data were collected: time posttransplant (days), creatinine (at the time of the biopsy, mg/dL), estimated GFR (eGFR, mL/min, based on the CKD-EPI equation), creatinine increase (mg/dL, delta between the baseline creatinine value and the value at the time of the biopsy), proteinuria (as mg/g). Creatinine predictions at 2 and 12 months indicate the creatine baseline values calculated as mean in the time range of 1.5–2.5 months and 11–14 months after biopsy, respectively. All data are presented in **Supplementary Table S2**.

### 2.3 Sample preparation

All samples were obtained at the time of the kidney graft biopsy. Blood was collected in serum tubes (S-Monovette, EDTA, Sarstedt, Nümbrecht, Germany). Within 60 min after the blood draw, serum samples were centrifuged (1,000×*g*, 15 min), and the supernatant was stored at − 80°C (cell freezing container, controlled cooling rate −1°C/min) until further analysis. The allograft biopsy was obtained as an 18-G needle biopsy and directly placed in RNAlater solution (Thermo Fisher, Waltham, MA, USA) at 4°C for a maximum of 2 h prior to RNA isolation. Adjacent tissue, such as fat or muscle, was removed, and the renal core of the biopsy was chopped into small pieces using a sterile scalpel. Next, the RNA was isolated using the column-based GenElute Total RNA Miniprep kit (Sigma Aldrich, St. Louis, MO, USA) and eluted in a total volume of 50–100 μL. Up to 150 μg of total RNA in 50–100 μL can be isolated in about 30 min. A median of 0.6 μg (0.3–94) RNA could be isolated from the biopsies. The isolated RNA was stored at − 80°C until further use.

### 2.4 Next-generation RNA sequencing

RNA quality was checked using the 4200 Tapestation (Agilent, Santa Clara, CA, USA). Only samples with RNA integrity numbers greater than 6 were used. TruSeq WGS libraries were prepared and sequenced with 25 M single reads per sample using the Illumina Next-Seq 500 sequencing platform at the Genomics Core Facility (University of Münster). Low-quality end trimming was performed using Trimmomatic version 0.39 (21). The minimum read length was 15 bp. The filtered reads were aligned to the Ensembl GRCh38 reference genome using HISAT2 version 2.1.0 (22) and were sorted using SAMtools version 1.9. We calculated the normalized read counts for each gene using DESeq2 (23) and used them as input for the Presto gene network analysis.

### 2.5 Presto gene network analysis and annotation

Predictive Stochastic Neighbor Embedding Tool for Omics (Presto) is a MatLab-based tool for analyzing coregulated gene networks in large omic datasets (24, 25). The gene expression matrix, as normalized read counts, serves as input. Prefiltering of highly variable genes is based on a coefficient of variation threshold. It was set for a total of about 2,000 genes. The expression values are normalized across all samples. The filtered genes are subjected to t-distributed stochastic neighbor embedding (t-SNE) that applies nonlinear dimensionality reduction and generates a two-dimensional scatterplot. Repeated runs change the macroanatomy of the plot but not the local clusters, as previously shown (24). The robustness of the method has been previously tested (24). K-means clustering is subsequently used to define distinct gene networks. The tool is available at https://github.com/saramcardle/PRESTO. All genes in all clusters were subjected to function annotation and term enrichment analysis using Metascape (26). It enables functional enrichment analysis (GO/KEGG/Reactome terms, canonical pathways). Statistically, the hypergeometric test and Benjamini–Hochberg *p*-value correction are used to identify terms that contain a greater number of genes in an input list than expected by chance. A subset of representative and significant terms is selected and displayed in the Presto graph.

### 2.6 Plasma proteomics

Serum proteins were measured using Olink technology (Sweden). It includes targeted proteomics based on the proximity extension assay. We used the panels “Inflammation I” and “Cardiometabolism”, each consisting of 92 predefined proteins (see Olink website). Each protein is targeted by two specific, oligonucleotide-labeled antibodies. Close antibody proximity enables DNA polymerization and extension. Measurements are done in triplicates. Proteins with low variance or under the detection threshold were removed. Protein concentrations are provided as relative expressions (normalized protein expression (NPX)). Frozen serum samples were thawed to an aliquot of 50 µL per patient on a 96-well plate. The plate was then stored at − 80°C for a short time until shipment on dry ice to Olink, Uppsala.

### 2.7 Correlation analyses

All correlation analyses used Spearman’s and subsequent hierarchical clustering (Spearman’s correlation, complete linkage). Similarity matrices correlated each protein versus all other proteins using Spearman’s correlation.

### 2.8 Statistics

The statistical tests used are indicated in the figure legends (^*^ *p* < 0.05; ^**^ *p* < 0.01; ^***^ *p* < 0.001; ^****^ *p* < 0.0001).

## 3 Results

We acquired a multi-omic dataset of 15 kidney transplant patients that includes bulk RNA next-generation sequencing of the allograft biopsy, targeted proteomics of 159 serum proteins, histological scoring, and clinical data. Since the serum samples were obtained at the time of the biopsy, RNA and protein measurements are time-matched. This cohort included for-cause biopsies with rejection and non-rejection diagnoses that were collected prior to therapy (**Supplementary Table S1**).

**Figure 1:**
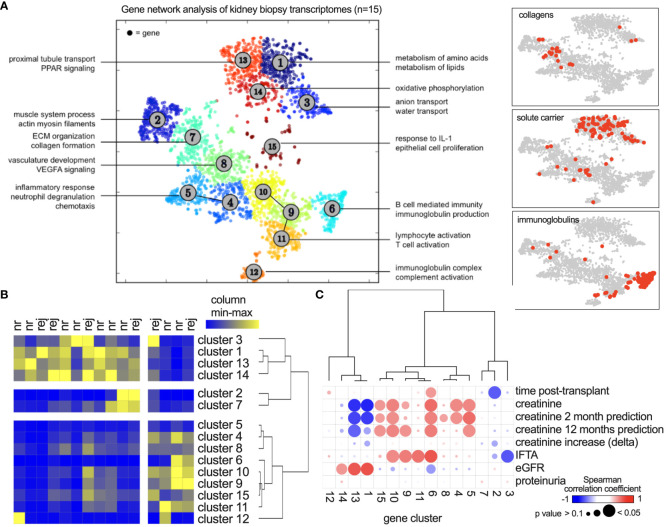
Coregulated gene network analysis of 15 kidney transplant biopsies. **(A)** After bulk RNA sequencing, Presto was applied using 2,613 highly variable genes. Each dot represents one gene, and clusters (networks) were defined using k means. Annotations show significantly enriched functions of each cluster. **(B)** Gene network activity in each biopsy shown as a heatmap with hierarchical clustering. Colors show relative expression from minimum (blue) to maximum (yellow) per column. nr, nonrejection; rej, any form of rejection according to the Banff classification. **(C)** Spearman’s correlation analysis of clinical parameters with gene network activity. “Creatinine increase” describes the increase compared to the patients’ baseline values. “Creatinine prediction” describes the association with creatinine values 2 or 12 months after the biopsy.

Coregulation analysis was applied to delineate RNA gene networks in the allograft transcriptome, resulting in 15 clusters (networks) with enrichment of specific functions (**Figure 1A**). The upper clusters (i.e., 1, 3, 13, 14) showed a tubule signature with metabolic and transport functions. The lower clusters included lymphoid and myeloid immune functions and extracellular matrix (ECM)-related genes (clusters 2, 7). Solute carriers were encoded in the former, and immunoglobulins and collagens in the latter (**Figure 1A**, right panels). Hierarchical clustering of each patient by gene cluster expression demonstrated metabolic, ECM, and immune phenotypes in the different patients to varying degrees (**Figure 1B**). They did not discriminate between nonrejecting and rejecting diagnoses. The first dendrogram branching point separated immune- and metabolism-related clusters (**Figure 1B**). Analysis of clinical parameters indicated that kidney function correlates positively with metabolic gene clusters and negatively with immune activity. Proteinuria did not show significant correlations (**Figure 1C**).

**Figure 2:**
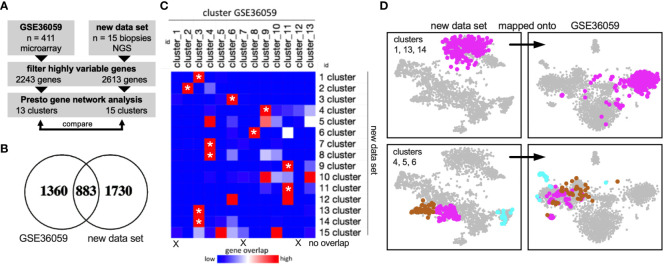
External validation of gene networks. **(A)** Workflow to compare the new data set with the published data set GSE36059 with 411 RTx biopsy transcriptomes. NGS, next-generation sequencing. **(B)** After filtering highly variable genes in both data sets independently using Presto, 883 common genes remained. **(C)** Gene overlap of each cluster (network) in both data sets. Asterisks mark gene clusters of the new data set that map predominantly (80% or more) to one specific cluster of the published data set. **(D)** Visualization of cluster genes in both data sets showing reproducible cluster assignments.

We next asked if these gene networks were reproducible. The published dataset GSE36059 contains 411 RTx for-cause biopsies from the University of Alberta, Canada. It includes bulk RNA sequencing data using microarray technology (**Figure 2A**). Here, using the same approach, 13 gene networks (clusters) could be delineated based on 2243 highly variable genes. Of these, 883 overlapped in both data sets (**Figure 2B**). Cluster comparison revealed a large extent of identical gene-cluster assignments (**Figure 2C**), including metabolic and immune gene clusters (**Figure 2D**). These data suggested that coregulated gene clusters in kidney allograft biopsies (RNA bulk sequencing) were largely similar across experiments, sequencing platforms, and transplant centers.

**Figure 3:**
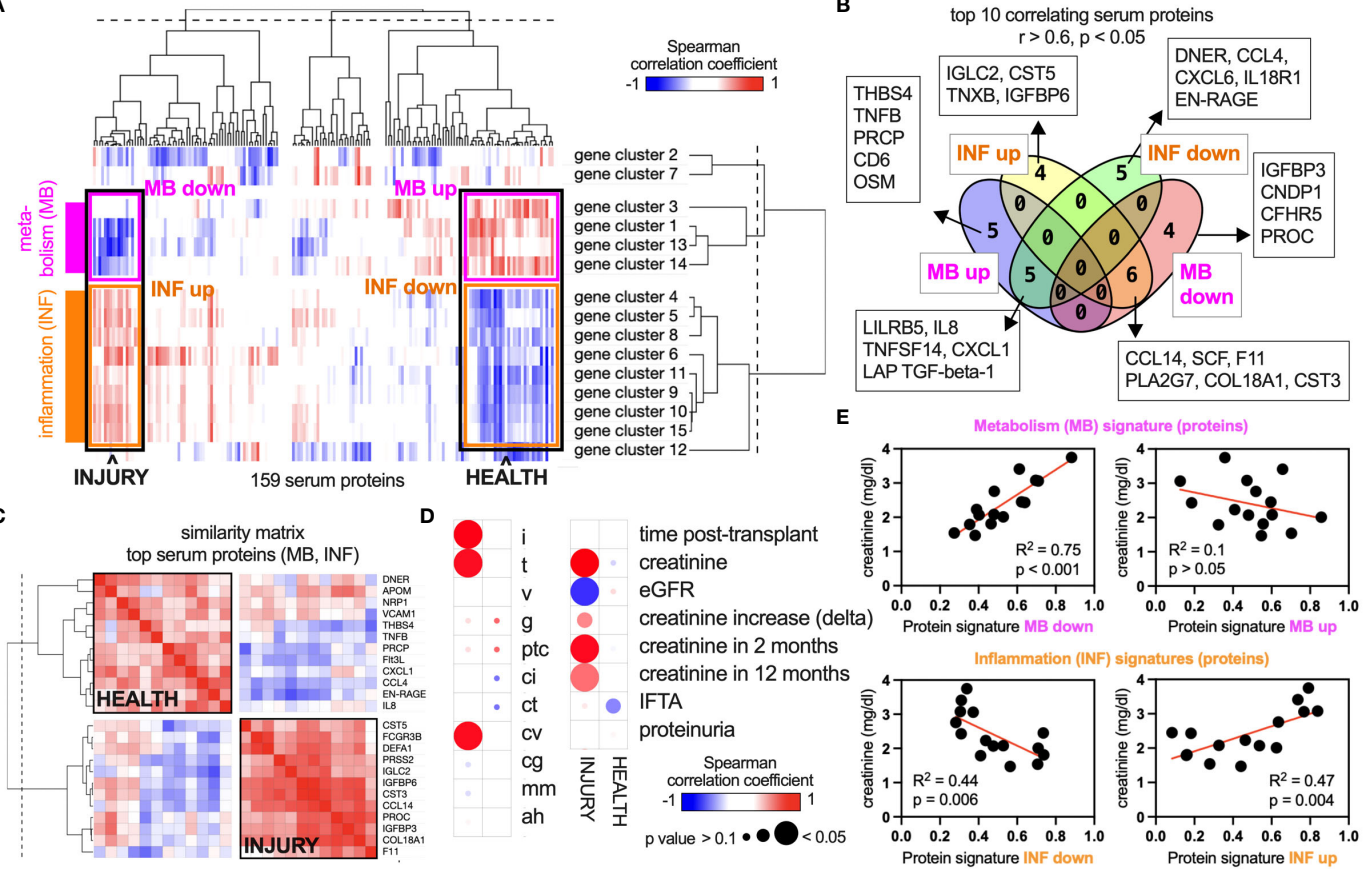
Unsupervised intercorrelation of the serum proteome and allograft gene networks. **(A)** An analysis of 159 protein concentrations in a biopsy-matched serum sample of each patient (*n* = 15) revealed correlations between these concentrations and the gene network activity within the corresponding biopsies. Columns, proteins; rows, gene networks. Blue, negative correlation; red, positive correlation. Networks 1, 3, 13, and 14 are related to metabolic functions (**Figure 1**) and are termed “metabolism” (MB). Networks 4, 5, 6, 8, 9, 10, 11, and 15 are related to immune activity and are termed “inflammation” (INF). **(B)** All proteins in the four categories “INF up/down” and “MB up/down” were filtered for a Spearman’s correlation coefficient > 0.6 or ≤ 0.6 and a *p*-value of < 0.05. **(C)** Proteins of the groups “HEALTH” (= MB up, INF down) and “INJURY” (= MB down, INF up) were selected, and a similarity matrix of the top 10 proteins was calculated (correlation of proteins with all other proteins). **(D)** MB and INF signatures (top 10 proteins each) were correlated with clinical parameters across 15 patients of the data set. **(E)** XY plots and regression analyses of correlations shown in **(D)**.

Our central hypothesis is that there are intricate links between allograft biology and other immune compartments in the body, including the blood serum. To correlate the graft gene landscape with serum proteins, we collected blood samples at the time of the graft biopsy and acquired targeted proteomic data. Hence, RNA and protein data were time-matched in all patients. Gene networks (i.e., mean gene expression of all genes in the network) were correlated with the concentration of 159 serum proteins across 15 patients in an unsupervised manner using a Spearman-based similarity matrix. After hierarchical clustering, two main axes emerged: The metabolic gene clusters (i.e., 1, 3, 13, 14) grouped together, and this axis was termed “metabolism” (MB) (**Figure 3A**). The second axis includes the immune-related gene clusters and is termed “inflammation” (INF). Both axes are opposed and correlate negatively or positively with the same serum proteins (**Figure 3A**). For example, some identified serum proteins correlate positively with MB and at the same time negatively with INF (right end of the axes), or the other way around (left end of the axes). The ends therefore define “INJURY” and “HEALTH” signatures in the serum that associate with a specific allograft transcriptome phenotype (**Figure 3A**). Of note, ECM-related gene clusters did not show a significant correlation. The top 10 serum proteins with the highest correlation coefficients for MB and INF up/down (*r* > 0.6) reaching significance (*p* < 0.05) are detailed in **Figure 3B**. Many INJURY- and HEALTH-related proteins showed a high interprotein correlation coefficient within the axis (MB or INF) and a negative correlation with the opposing axis (**Figure 3C**). Correlating the glomerular filtration rate (GFR) and serum creatinine with MB (up/down) and INF (up/down) (i.e., mean of all protein concentrations in each signature) confirmed a connection to allograft function (**Figures 3D, E**). Proteinuria and transplant duration were independent in this analysis.

**Figure 4:**
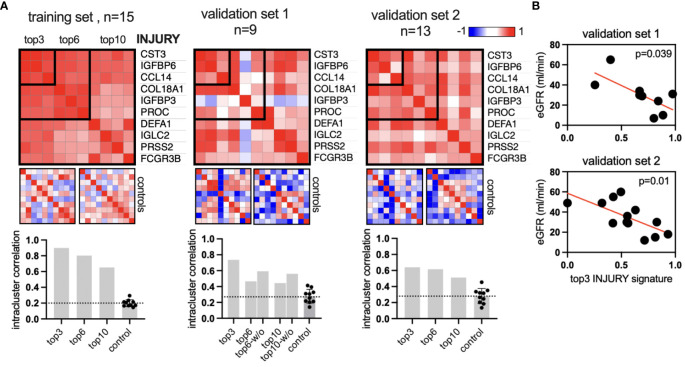
Validation of the INJURY signature in two independent cohorts. **(A)** Similarity matrices (Spearman’s correlation of all proteins) of top 10, top 6, and top 3 proteins of the INJURY signature from **Figure 3**. Left, the training set from **Figure 3**; center and right, the two independent validation data sets with *n* = 9 and 13 patients, respectively. The intracluster correlation was calculated and plotted in the bar graphs. Each control includes 10 sets with 10 random proteins, two of which are visualized as example heatmaps. The bar graph in validation set 1: “w/o” shows the correlation coefficient without the outlier IGFBP3. **(B)** The top 3 injury signatures were plotted against the glomerular filtration rate (GFR) in both validation data sets.

Next, we sought to validate the INJURY protein signature in two independent validation cohorts (27). Serum proteins were measured in nine and 13 kidney transplant patients using the same approach. Notably, the ratio of nonrejection, TCMR, and ABMR varied between the data sets (**Supplementary Table S1**). The validation data sets featured only proteomic data. For a positive validation, we assume the protein signature to (1) show a higher intrasignature correlation compared to a random set of proteins and (2) associate with allograft function. The INJURY signature includes the top positively correlating proteins, as shown in **Figure 3**. We analyzed the top 3, top 6, and top 10 proteins (descending order by correlation coefficient: CST3, IGFBP6, CCL14, COL18A1, IGFBP3, PROC, DEFA1, IGLC2, PRSS2, and FCGR3B). The intrasignature correlation was determined by averaging the correlation coefficient of all pairwise protein correlations in the signature. Ten sets of 10 random proteins in each data set were used as controls. This analysis concludes that the top 10, top 6, and top 3 INJURY signatures show higher intrasignature correlation compared to controls (**Figure 4A**). The top 3 signatures showed the highest interprotein correlation. It was then correlated with kidney function, which confirmed a significant association in both data sets (**Figure 4B**). These results suggest that the INJURY signature defines serum proteins that are linked both to local allograft inflammation (on the transcriptome level) and renal function.

## 4 Discussion

Data-driven experiments contrast many reductionist analyses in RTx (20) that link histology-based diagnosis with target molecules or outcomes (28–33). This study diverged from using the gold standard (Banff histological scoring) for clinical classification. Instead, we applied next-generation RNA sequencing, known for its heightened sensitivity in detecting graft injury compared to histology, thus circumventing biases associated with imperfect gold standards (34, 35). In this line, a recent study compared the allograft transcriptome to plasma donor-derived cell-free DNA (36). This work was based on the commercial “Molecular Microscope Diagnostic System” (37) that offers a supervised framework of RNA allograft signatures underlying the Banff diagnosis. Furthermore, our study avoided analyzing specific molecular targets, opting instead for a larger panel of serum proteins for hypothesis-free correlation analysis. This approach, akin to previous studies in CKD (38) and RTx (27), unveiled distinct protein modules associated with kidney function. Our findings suggest that these serum-derived modules may directly reflect various patterns of allograft homeostasis and injury.

This finding has several implications. It highlights a biological continuum extending from the allograft to the blood serum. Over the years, liquid biopsies have been explored through various blood signatures (28, 29, 39, 40). Our data confirm the suitability of blood serum as a medium for RTx biomarkers. In addition, the presence of two axes indicates that both metabolic health and immune activity in the allograft are accompanied by specific changes in the serum. Thus, not only inflammation but also immune quiescence and graft homeostasis are detectable as distinct signatures in the blood. In our analysis, graft ECM activity was not linked to these axes. However, this finding is not conclusive, as the selection of measured proteins specifically targeted inflammation and the number of biopsies with high-grade fibrosis was low. It was surprising to find serum proteins that correlate only with one axis or with both. For example, the INJURY signature includes six proteins that correlate with both axes (“MB down” and “INF up”), while four proteins only correlate with “INF up” and four proteins only correlate with “MB down”. Hence, serum biomarkers might reflect different dynamics and phases of allograft injury. These results propose the concept of a serum-allograft axis as a potential new framework for developing peripheral biomarkers centered around the allograft. This approach could prove invaluable for detecting subclinical allograft injury (33) or monitoring renal regeneration. Integrating systemic responses directly linked to allograft biology could help overcome the current challenges of biomarker studies (41). Due to the patient and protein selection in this study, the derived INJURY signature predominantly reflects histological inflammation and tubulitis (**Figure 3D**). However, broadening the scope to include other pathologies and omic data could unveil novel serum–graft interrelations, potentially leading to actionable biomarkers. It is probable that other body fluids, such as urine, are an integrative part of systemic responses during RTx.

The INJURY signature includes the insulin-like growth factor-binding proteins (IGFBP)6 and IGFBP3. A previous study found that CKD is associated with a serum increase of IGFBP6, and renal transplantation largely reverses this finding (42). Subsequent investigations added a role of IGFBP6 and IGFBP3 in other kidney diseases such as diabetic nephropathy, glomerulosclerosis, and IgA nephropathy (43). IGFBP3 was shown to modulate the TGF-β/BMP-7 signaling pathway in podocyte apoptosis (44) and inversely correlate with kidney function (45). However, our data did not uncover an association of the INJURY signature with proteinuria and glomerulitis, excluding a podocyte-dependent phenotype. The top 3 INJURY signatures contained CCL14. It was shown to predict renal nonrecovery in acute kidney injury (46), but evidence of kidney transplantation is missing. The HEALTH signature features the chemokines CXCL1, CXCL8 (IL-8), CCL4 (MIP-1β), and lymphotoxin alpha (TNFB). The association with renal inflammation is surprising (47–49), suggesting that the metabolic health signature (MB up, **Figure 3**) cannot be interpreted as the homeostatic state of the allograft. We hypothesize that the INJURY signature represents one distinct injury pattern that is independent of the serum regulation of these chemokines. As a consequence, the HEALTH signature aggregates all other phenotypes with opposing traits compared to INJURY, including other types of inflammation and homeostasis. Our set of patients included only for-cause biopsies, i.e., allograft pathologies necessitating invasive diagnostics. The inclusion of protocol biopsies with pristine allografts would be required to describe a full axis from homeostasis to injury.

The study has more limitations to be considered. The high number of variables and low number of patients pose a risk for overfitting. Hence, we used broad categories like “nonrejection/rejection” and “health/injury” and refrained from a more granular analysis. Larger studies are needed to validate our results and improve specificity and sensitivity. Second, many independent variables, such as medication and comorbidity, were not accounted for. Moreover, the resulting signatures depend on the input variables (i.e., genes and proteins) and clustering methods (50). We used a targeted approach that measures a preselected, inflammation-centered panel of proteins. The analysis of the full proteome would be insightful to develop a comprehensive picture of the allograft-serum proteome axis. Finally, correlation does not imply causality. The biological role of the target proteins and the cause of their high intracluster correlation remain to be established.

In conclusion, this study suggests that a serum-allograft axis is a prominent feature in renal transplantation, offering a promising framework for developing allograft-linked serum biomarkers.

## Data availability statement

The data presented in the study are available in supplemental table 2. The raw data are deposited in the GEO repository, accession number GSE269931.

## Ethics statement

The studies involving humans were approved by Ethik-Kommission der Ärztekammer Westfalen-Lippe und der Universität Münster”; No 2011-400-f-S and 2019-337-f-S. The studies were conducted in accordance with the local legislation and institutional requirements. The participants provided their written informed consent to participate in this study.

## Author contributions

KB: Conceptualization, Data curation, Formal analysis, Funding acquisition, Investigation, Methodology, Project administration, Resources, Software, Supervision, Validation, Visualization, Writing – original draft, Writing – review & editing. RR: Data curation, Formal analysis, Writing – original draft. PS: Data curation, Formal analysis, Writing – original draft. VVM: Formal analysis, Methodology, Writing – original draft. BH: Formal analysis, Methodology, Writing – original draft. GG: Methodology, Resources, Writing – original draft. UJ: Methodology, Resources, Writing – original draft. DB: Methodology, Resources, Writing – original draft. HP: Writing – review & editing. SR: Methodology, Resources, Writing – original draft, Writing – review & editing.
