# Supplementary material for: Unveiling systemic responses in kidney transplantation: interplay between the allograft transcriptome and serum proteins
Source: Front Immunol. 2024 Jul 16;15:1398000. doi: 10.3389/fimmu.2024.1398000 (PMC11286594; doi:10.3389/fimmu.2024.1398000)
Supplement: Supplementary file 1 [file Table_1.docx]

|  | **training set** | **validation set 1** | **validation set 2** |
| --- | --- | --- | --- |
| **samples** | 15 | 9 | 13 |
|  |  |  |  |
| **patient characteristics** |  |  |  |
| -age (years, median) | 47 (23-71) | 40 (22-80) | 44 (28-62) |
| -time post-transplant (median, days) | 1746 (21-7823) | 2250 (7-5444) | 1021 (85-3915) |
| -creatinine (median, mg/dl) | 2.39 (1.47-3.75) | 3.15 (1.18 – 7.29) | 2.40 (1.05-5.21) |
| -eGFR (median, ml/min) | 32 (17-66) | 30 (10-65) | 34 (12-60) |
| -donor specific antibodies (DSA) | 2/15 | 3/9 | 5/13 |
|  |  |  |  |
| **histological diagnosis** |  |  |  |
| -non-rejecting | 9/15 | 3/9 | 4/13 |
| -antibody-mediated rejection | 0/15 | 2/9 | 4/13 |
| -T cell-mediated rejection | 4/15 | 4/9 | 3/13 |
| -Mixed rejection | 2/15 | 0/9 | 2/13 |
|  |  |  |  |
| -Interstitial fibrosis and tubular atrophy (IFTA)  none  low  medium  high | 3/15  8/15  3/15  1/15 | 4/9  0/9  3/9  2/9 | 1/13  9/13  3/13  0/13 |
|  |  |  |  |
| **Banff scoring** | in 9/15 biopsies | in 9/9 biopsies | in 13/13 biopsies |
| i (inflammation) | 0.66 (0-1) | 0.77 (0-1) | 1.07 (0-2) |
| t (tubulitis) | 0.44 (0-1) | 0.55 (0-1) | 0.61 (0-2) |
| g (glomerulitis) | 0.11 (0-1) | 0.33 (0-1) | 1.0 (0-2) |
| ptc (peritubular capillaritis) | 0.11 (0-1) | 0.33 (0-1) | 0.61 |
| ci (interstitial fibrosis) | 1.0 (0-2) | na | na |
| ct (tubular atrophy) | 1.0 (0-2) | na | na |
| cv (vascular fibrous intimal thickening) | 0.55 (0-2) | na | na |
| cg (basement membrane double contours) | 0.11 (0-2) | na | na |
| mm (mesangial matrix expansion) | 0.11 (0-2) | na | na |
| ah (arterial hyalinosis) | 1.0 (0-3) | na | na |
|  |  |  |  |
| **immunosuppression** |  |  |  |
| -tacrolimus | 12/15 | 8/9 | 11/13 |
| -everolimus | 3/15 | 1/9 | 1/13 |
| -cyclosporine A | 1/15 | 0/9 | 2/13 |
|  |  |  |  |
| **renal disease** |  |  |  |
| -Glomerulonephritis | 3/15 | 2/9 | 4/13 |
| -FSGS | 1/15 | 1/9 | 1/13 |
| -Goodpasture syndrome | 0/15 | 1/9 | 0/13 |
| -Polycystic kidney disease | 2/15 | 1/9 | 1/13 |
| -Congenital hypoplasia | 0/15 | 1/9 | 1/13 |
| -Nephrosclerosis | 2/15 | 1/9 | 1/13 |
| -Atypical HUS | 1/15 | 0/9 | 1/13 |
| -Ureteral stenosis | 0/15 | 0/9 | 1/13 |
| -Others | 6/15 | 2/9 | 3/13 |
